# Agricultural abundance, dietary shortfall: national determinants of daily fruit and vegetable intake in Türkiye National Nutrition and Health Survey

**DOI:** 10.1186/s41043-026-01310-0

**Published:** 2026-04-13

**Authors:** Merve Korkmaz, Zeynep Begüm Kalyoncu Atasoy

**Affiliations:** 1 Faculty of Health Sciences, Department of Nutrition and Dietetics, Istanbul Aydin University, Istanbul, Türkiye; 2 Gülhane Faculty of Health Sciences, Department of Nutrition and Dietetics, University of Health Sciences, Ankara, Türkiye

**Keywords:** Fruit, Vegetables, Food consumption, Nutrition survey, Türkiye

## Abstract

**Background:**

Türkiye is one of the world’s leading producers of fruits and vegetables (F&V). Yet, findings from the Türkiye Nutrition and Health Surveys (TNHS-2010 and TNHS-2017) reveal a 24.3% decline in consumption over this period. This discrepancy between agricultural capacity and dietary intake highlights the need to better understand factors associated with consumption. This disconnect between agricultural abundance and dietary intake underscores the need to understand the factors influencing F&V consumption at the population level.

**Methods:**

This cross-sectional secondary analysis used data from the nationally representative TNHS-2017. The analytic sample included 12,406 individuals aged ≥ 15 years who completed the Food Frequency Questionnaire. Anthropometric measurements were obtained by trained dietitians in Family Health Centers across all 81 provinces of the country; pregnant and lactating women were excluded. F&V intake was categorized as daily versus non-daily, and freshly squeezed fruit juice as never, occasional, or daily. Binary logistic regressions were used to identify factors associated with daily F&V intake, and multinomial logistic regression was used to examine factors associated with juice consumption.

**Results:**

Daily F&V intake was observed in 38.6% of participants. Higher odds of daily intake were seen among women (aOR 1.58, 95% CI 1.44–1.73), older adults, individuals with greater physical activity, and dietary supplement users, whereas higher food insecurity was inversely associated with daily intake (aOR per unit 0.90, 95% CI 0.86–0.93). Regional disparities were pronounced, with higher odds in the Mediterranean and Middle East Anatolia and lower odds in several inland and northeastern regions relative to the Aegean. BMI and body roundness index were not independently associated with intake after adjustment. For freshly squeezed fruit juice, occasional consumption was more likely among higher-educated groups and supplement users and less likely among smokers and food-insecure individuals. Daily juice consumption was rare.

**Conclusions:**

In Türkiye, daily F&V intake was associated with sociodemographic, regional, behavioral, and food security factors rather than body size indicators. These findings provide nationally relevant evidence to support strategies aimed at improving fruit and vegetable consumption, particularly by addressing affordability, regional disparities, and food insecurity, while clarifying the limited role of juice.

**Supplementary Information:**

The online version contains supplementary material available at 10.1186/s41043-026-01310-0.

## Introduction

 Insufficient intake of fruits and vegetables (F&V) compromises multiple physiological systems and is a major, yet preventable, contributor to noncommunicable disease (NCD) mortality and nutritional inequity worldwide [1]. Rich in dietary fiber, potassium, antioxidants, and anti-inflammatory phytochemicals, F&V consumption help regulate blood pressure, improve lipid profiles, support insulin sensitivity, and protect against oxidative stress that are central to the prevention of cardiovascular diseases, type 2 diabetes, and certain cancers [2–4]. However, despite decades of global dietary guidance, suboptimal intake has thus been recognized as a significant global dietary risk. Most populations fall short of World Health Organization’s minimum target of ≥ 400 g/day, especially in low- and middle-income countries (LMICs) [5, 6].

In 2021, inadequate fruit and vegetable (F&V) consumption was linked to an estimated 2.6 million deaths and 64.5 million disability-adjusted life years (DALYs) globally [7]. According to the Global Burden of Disease (GBD) Study 2021, low fruit intake accounted for 1.7 million deaths and 43.8 million DALYs, while low vegetable intake accounted for 0.9 million deaths and 20.7 million DALYs. Across both dietary risks, cardiovascular diseases accounted for the largest share of attributable mortality (83.7% for low fruit intake and 79.3% for low vegetable intake), with additional contributions from diabetes and kidney diseases and cancers [7].

These patterns reflect not only individual dietary choices, but also systemic failures in ensuring equitable access to affordable, nutritious food [8]. Inadequate F&V intake is a marker of structural nutrition inequity, often coexisting with other diet-related disparities among socioeconomically disadvantaged and minority groups and contributing to disproportionate chronic disease burdens [9]. These disparities persist globally and are now compounded by economic, environmental, and political stressors that influence food choices [10].

Türkiye exemplifies a striking disconnect between its exceptionally high production and historically plant-rich food culture [11, 12] and emerging signs of nutritional backsliding. Türkiye shows substantial regional heterogeneity in food systems and health outcomes. Fruit and vegetable production is spatially concentrated, reflecting regional specialization in supply conditions and market availability [13]. Mortality indicators also vary geographically, with TurkStat reporting subnational differences in crude death rates and cause-of-death patterns across provinces [14, 15]. Against this backdrop, examining how daily fruit-and-vegetable consumption varies across Türkiye’s statistical regions may help inform region-sensitive nutrition policies and monitoring efforts. Türkiye’s updated Food-Based Dietary Guidelines (TÜBER 2022) explicitly promote a plant-forward eating pattern, recommending at least five daily servings of fruits and vegetables, with portion guidance by age and sex groups [12]. Yet, according to the Türkiye Nutrition and Health Survey (TNHS), mean daily F&V intake among adults declined from ~ 540 g/day in 2010 to ~ 415 g/day in 2017, reflecting a 23.1% reduction in only seven years [16]. This decline occurred despite continued growth in domestic supply: external statistics based on TurkStat data indicate that total fruit and vegetable production increased from approximately 42.6 million tons in 2010 (≈ 16.5 million tons fruit and ≈ 26.1 million tons vegetables) to 51.6 million tons in 2017 [17, 18]. Hence, the observed intake decline contradicts public health targets and exposing an implementation gap that may reflect rising economic hardship, shifting food preferences, or supply chain constraints even in a country with robust agricultural production. In this context, it is increasingly important to examine the sociodemographic and behavioral factors associated with fruit and vegetable consumption, particularly in settings like Türkiye where agricultural abundance does not necessarily translate to adequate intake. Studies exploring these determinants remain limited, and few have utilized population-representative data. To address this gap, this study provides a comprehensive analysis of F&V consumption patterns among individuals aged 15 and older, using nationally representative Türkiye Nutrition and Health Survey (TNHS) 2017 data. To address this gap, we analyzed nationally representative data from the Türkiye Nutrition and Health Survey (TNHS) 2017 to characterize patterns of fruit and vegetable consumption among individuals aged ≥ 15 years. We further examined sociodemographic and behavioral correlates of daily fruit-and-vegetable consumption and assessed whether prevalence differed across Türkiye’s statistical regions. This evidence may help inform targeted, equity-oriented nutrition policies and monitoring efforts in Türkiye, while offering comparative insights relevant to international settings, including efforts to address nutrition disparities in other middle- and high-income countries.

## Methods

### Study design and data source

This study is based on secondary analyses of data from the Türkiye Nutrition and Health Survey 2017 (TNHS-2017), a nationally representative, cross-sectional survey of the non-institutionalized population aged ≥ 15 years in Türkiye, conducted by the Ministry of Health in collaboration with universities. TNHS-2017 was implemented within the Türkiye Healthy Nutrition and Active Life Program and aligned with the 10th Development Plan (2014–2018) [19]. The survey provides periodic information on dietary intake, nutritional status, and related health indicators and includes comprehensive dietary, sociodemographic, health, and behavioral data from approximately 13,000 participants. Prior to nationwide fieldwork, the study instruments, including computer-assisted personal interviewing (CAPI) modules programmed in LimeSurvey [20], were piloted in Ankara and Gaziantep to test logistics and refine data-entry procedures. Main fieldwork was carried out between February and August 2017 across all 81 provinces of Türkiye. Trained dietitians based in Family Health Centers conducted interviews, administered questionnaires, and obtained anthropometric measurements according to standardized protocols [16]. To date, four national nutrition and health surveys have been conducted in Türkiye—1974, 1984, 2010, and 2017—each aiming to monitor the population’s nutritional status and identify prevailing public health challenges [21]. The present analysis uses TNHS-2017 to examine fruit and vegetable consumption and its sociodemographic and behavioral correlates.

### Sample

The sampling design was developed by the Turkish Statistical Institute (TurkStat) using a three-stage, stratified cluster approach to ensure coverage across Türkiye’s regions and urban–rural areas. In the first stage, residential addresses were grouped into clusters of roughly 100 households, and 2,400 clusters were randomly chosen with probability proportional to size. In the second stage, 10 addresses were systematically selected from each cluster. In the third stage, one eligible individual was randomly drawn from each selected household using the Ministry of Health’s Family Medicine Information System. Sampling frames were derived from the 2017 Address-Based Population Registration System and the National Address Database. Participants were invited to attend their Family Health Center in a fasting state (≥ 8 h). Those unable to attend were re-invited, and, after three missed appointments, home visits were conducted to maximize participation. According to the TNHS 2017 report, 12,984 participants completed the survey, including the Food Frequency Questionnaire (FFQ). For the present study, pregnant women (*n* = 167) and lactating women (*n* = 414) were excluded. As 3 participants belonged to both categories, these exclusions were overlapping, yielding 578 unique exclusions in total. The final analytic sample comprised 12,406 participants with complete Food Frequency Questionnaire (FFQ) data, including 6,583 women and 5,823 men, and was classified into three age groups: adolescents (15–18 years, *n* = 526), adults (19–64 years, *n* = 9,620), and older adults (≥ 65 years, *n* = 2,260) [16].

Ethical approval for the original survey, including written informed consent from all participants, was obtained from the Zekai Tahir Burak Hospital Ethics Committee (Decision No. 2011-KAEK-19). Approval for this secondary analysis was granted by the Istanbul Aydın University Ethics Committee (Decision No. 86/2024).

### Determining fruit and vegetable consumption

In TNHS-2017, participants aged 15 years and older completed a FFQ assessing how often they consumed specific foods and beverages over the past month. For the present analysis, FFQ data were used to evaluate fruit and vegetable consumption. The survey collected intake frequency for multiple items, including total fruit, total vegetables, leafy greens, other fresh vegetables (e.g., leeks, cabbage), tomatoes, green peppers, corn, mushrooms, other fresh fruits, frozen fruits/vegetables, dried fruits/vegetables, citrus fruits, and freshly squeezed fruit juice. Response options were: “Never,” “Less than once per month,” “1–3 times per month,” “Once per week,” “2–3 times per week,” “4–5 times per week,” “Every day,” and “Don’t know / No response.”

For this study, daily fruit-and-vegetable (F&V) consumption was defined using the “total fruit” and “total vegetable” items. The ‘total fruit’ and ‘total vegetable’ items are overall summary frequency questions and are not intended to equal the sum of subtype frequencies; therefore, daily F&V status was defined using these total items. Participants selecting “Every day” for both items were classified as daily consumers; all others—including those reporting daily intake for only one of the two (fruit or vegetables)—were classified as non-daily consumers. This strict definition was selected to capture a clear indicator of habitual daily consumption of both food groups that is easy to interpret for policy monitoring and subgroup comparisons using frequency data. Portion size/grams were not available from these FFQ items; therefore, guideline adherence (≥ 400 g/day) could not be assessed. Similarly, freshly squeezed fruit juice consumption was categorized into three groups: never (non-consumers), occasional (all frequencies other than never and every day), and daily (“every day”). Responses coded as “don’t know / no response” or missing were excluded from analyses. Because the primary outcomes were derived from FFQ-based consumption frequency variables rather than from energy intake estimated using 24-hour dietary recall data, under-reporting was not formally assessed using energy intake–based methods. Participants were included according to the completeness of the relevant FFQ data, and cases with missing values in covariates included in regression models were excluded from the respective analyses. To examine the robustness of this categorization, supplementary sensitivity analyses were also conducted using an alternative binary definition based on consuming fruit and vegetables more than once per week versus once per week or less. These findings are presented in **Supplementary Tables S1a and S1b**. Freshly squeezed fruit juice consumption was modeled as a three-category outcome using multinomial logistic regression with never as the reference category (occasional vs. never; daily vs. never).

### Assessment of covariates

Covariates were selected to control for potential confounders of F&V intake. Sociodemographic variables included age (continuous; also categorized as 15–18, 19–64, and ≥ 65 years), sex, marital status, education level, household economic situation, and geographic region. Education level was categorized as illiterate, literate (no formal schooling), primary school, middle school, high school or equivalent, and university. Household economic situation was categorized into four self-reported levels reflecting perceived ability to meet monthly expenses (from “more than adequate” to “insufficient”), as detailed in Table 1. Geographic region was classified using the NUTS-1 system (12 statistical regions) (e.g., Istanbul, Aegean, Central Anatolia, etc.), with all region categories shown in Table 1 [22]. Health and lifestyle characteristics included presence of physician-diagnosed chronic disease (yes/no), smoking status (never, former, current), dieting status (no—weight is fine; no—need to lose weight; no—need to gain weight; yes), vegetarian status, dietary supplement use, and physical activity level (PAL, continuous). Food insecurity was assessed using the Food Insecurity Experience Scale (FIES), developed by the Food and Agriculture Organization (FAO), and analyzed as a continuous variable, with higher scores indicating greater insecurity. The FIES is an experience-based instrument consisting of eight items that evaluate individuals’ or households’ access to adequate quality and quantity of food, providing a standardized measure of the access dimension of food security. In addition to the continuous score, categorical thresholds were applied to classify participants as food secure, moderately food insecure, or severely food insecure [23].

Anthropometric indicators included body mass index (BMI; kg/m²), waist-to-hip ratio (WHR; ≥0.90 for men and ≥ 0.85 for women indicating elevated risk in descriptive reporting), basal metabolic rate (BMR), and total energy expenditure (TEE). The Body Roundness Index (BRI) was additionally calculated in the present study as an alternative marker of adiposity. BRI incorporates waist circumference and height to estimate body shape and body fat distribution, providing a more direct indicator of central adiposity than BMI. The formula is as follows:

$$\begin{aligned}&\mathrm{BRI}=364.2-365.5\\ &\quad\times\sqrt{1-\frac{(\text{Waist Circumference (cm)})^2}{(2\pi)^2\times (o.5\times \mathrm{Height} (\mathrm{cm}))^2}}\end{aligned}$$

BRI has been validated against body fat percentage and cardiometabolic risk factors and shown to predict cardiovascular disease and diabetes more accurately than BMI in some populations [24].

### Statistical analyses

The Kolmogorov–Smirnov test was applied to evaluate the normality of data distribution. Between-group comparisons of non-normally distributed continuous variables were performed using the Mann–Whitney U test, and correlations were examined with Spearman’s rank-order correlation (rho). Associations between categorical variables were assessed with the Pearson chi-square test or Fisher’s exact test with Monte Carlo correction, and multiple comparisons were evaluated using the Bonferroni-corrected Z test. Psychometric properties of the Food Insecurity Experience Scale (FIES) were assessed using an item response theory measurement model, specifically the single-parameter logistic Rasch model. In line with FAO guidance, Rasch reliability coefficients above 0.70 were considered acceptable for an 8-item scale and above 0.60 for a 7-item scale. Conditional independence and dimensionality were examined using item residual correlations, with values ≥ 0.40 between item pairs interpreted as indicating local dependence [25]. Binary logistic regression analysis was used to examine the adjusted associations of daily fruit and vegetable consumption and freshly squeezed fruit juice consumption with moderate food insecurity. In this model, the dependent variable was food insecurity category, coded as moderate versus mild according to the seven-item Rasch-calibrated FIES model, and age, sex, and income status were included as covariates. Univariate and multivariable binary logistic regression analyses were performed to identify factors associated with daily fruit and vegetable consumption. In addition, multinomial logistic regression analysis was used to examine factors associated with freshly squeezed fruit juice consumption, categorized as never (reference), occasional (all frequencies other than never and daily), and daily. Continuous variables are reported as median (minimum–maximum), and categorical variables as frequency (percentage). Statistical significance was set at *P* < 0.05. Analyses were conducted using IBM SPSS Statistics version 23. The TNHS-2017 dataset included calibrated survey weights generated by the original survey team. During the preparation of this manuscript, the authors used ChatGPT-5 (OpenAI, San Francisco, CA, USA) to assist with language editing and data visualization. After using this tool, the authors carefully reviewed and revised all content and take full responsibility for the final version of the manuscript.

## Results

### Participant characteristics

A total of 12,406 participants aged ≥ 15 years were included (46.89 ± 17.72 years; 53.1% women); descriptive characteristics by daily vs. non-daily fruit and vegetable (F&V) consumption are summarized in Table 1. Daily fruit and vegetable consumption was observed in 38.6% of the study population. Daily F&V consumers were older than non-daily consumers (median 49 vs. 43 years, *P* < 0.001) and more often women (59.7% vs. 49.0%, *P* < 0.001). Education and marital status distributions differed by consumption status (both *P* < 0.001). Regional variation was pronounced (*P* < 0.001), with higher shares of daily consumers in the Aegean/Mediterranean and lower shares in Northeast/Central Anatolia (Table 1). Non-daily consumers had higher food insecurity (FIES; *P* < 0.001). Current smoking was less prevalent among daily consumers (26.9% vs. 35.6%, *P* < 0.001), while supplement use was more common (11.6% vs. 8.9%, *P* < 0.001) and physical activity level slightly higher (median PAL 1.75 vs. 1.72, *P* < 0.001). Daily consumers showed marginally higher BMI and BRI (both *P* < 0.001), no difference in WHR (*P* = 0.339), and modestly lower BMR and total energy expenditure (both *P* < 0.001). In supplementary sensitivity analyses using an alternative threshold of more than once-weekly fruit and vegetable consumption, the overall pattern and direction of associations were broadly consistent with the main analyses, supporting the robustness of the primary categorization (Supplementary Tables S1a–S1b).

**Table 1:** Comparison of anthropometric, sociodemographic, health, lifestyle characteristics and food insecurity scores by daily fruit and vegetable consumption status

Sociodemographic Characteristics	Non-Daily Consumers Median (IQR) /%; Mean ± SD (*n* = 7664)	Daily Consumers Median (IQR) /% (*n* = 4742)	Total	Test Statistic	*P* ^x^	ES [95% CI]
**Age (years)**	43 (15–105) / 45.5 ± 17.8	49 (16–99) / 49.14 ± 17.35	45 (15–105) / 46.89 ± 17.72	15911059.500	**< 0.001** ^**x**^	0.211 [0.174: 0.247] ^d^
**Age group**						
Adolescent^1^	383 (5%)^a^	143 (3%)^b^	526 (4.2%)	53.902	**< 0.001** ^**y**^	0.066 [0.049: 0.083]^V^
Adult^2^	6001 (78.3%)^a^	3619 (76.3%)^b^	9620 (77.5%)			
Older Adult^3^	1280 (16.7%)^a^	980 (20.7%)^b^	2260 (18.2%)			
**Sex**						
Women	3752 (49%)	2831 (59.7%)	6583 (53.1%)	135.782	**< 0.001** ^**y**^	0.105^φ^
Men	3912 (51%)	1911 (40.3%)	5823 (46.9%)			
**Education status**						
Illiterate	734 (9.6%)	464 (9.8%)	1198 (9.7%)	53.453	**< 0.001** ^**y**^	0.066 [0.052: 0.083] ^V^
Literate	295 (3.8%)^a^	218 (4.6%)^b^	513 (4.1%)			
Primary school	2404 (31.4%)^a^	1718 (36.2%)^b^	4122 (33.2%)			
Primary school graduate	153 (2%)	113 (2.4%)	266 (2.1%)			
Middle school	801 (10.5%)^a^	427 (9%)^b^	1228 (9.9%)			
Middle school graduate	302 (3.9%)	183 (3.9%)	485 (3.9%)			
High school equivalent	1627 (21.2%)^a^	842 (17.8%)^b^	2469 (19.9%)			
University	1348 (17.6%)	777 (16.4%)	2125 (17.1%)			
**Marital status**						
Never married	1532 (20%)^a^	635 (13.4%)^b^	2167 (17.5%)	100.229	**< 0.001** ^**xx**^	0.089 [0.072: 0.109] ^V^
Married	5125 (66.9%)^a^	3451 (72.8%)^b^	8576 (69.1%)			
Widowed	683 (8.9%)^a^	487 (10.3%)^b^	1170 (9.4%)			
Divorced	293 (3.8%)^a^	148 (3.1%)^b^	441 (3.6%)			
Separated	31 (0.4%)	21 (0.4%)	52 (0.4%)			
**Income status**						
Income level 1^4^	1586 (20.7%)	1049 (22.1%)	2635 (21.2%)	11.134	**0.025** ^**y**^	0.030 [0.019: 0.054] ^V^
Income level 2^5^	2006 (26.2%)	1230 (25.9%)	3236 (26.1%)			
Income level 3^6^	2727 (35.6%)	1728 (36.4%)	4455 (35.9%)			
Income level 4^7^	1277 (16.7%)^a^	702 (14.8%)^b^	1979 (16%)			
Do not know	68 (0.9%)	33 (0.7%)	101 (0.8%)			
**NUTS regions**						
Istanbul	1106 (14.4%)	641 (13.5%)	1747 (14.1%)	165.360	**< 0.001** ^**xx**^	0.116 [0.100: 0.135] ^V^
Western Marmara	521 (6.8%)^a^	227 (4.8%)^b^	748 (6%)			
Aegean	1261 (16.5%)^a^	894 (18.9%)^b^	2155 (17.4%)			
Eastern Marmara	882 (11.5%)^a^	399 (8.4%)^b^	1281 (10.3%)			
Western Anatolia	764 (10%)	489 (10.3%)	1253 (10.1%)			
Mediterranean	931 (12.1%)^a^	767 (16.2%)^b^	1698 (13.7%)			
Central Anatolia	461 (6%)^a^	187 (3.9%)^b^	648 (5.2%)			
Western Black Sea	485 (6.3%)^a^	345 (7.3%)^b^	830 (6.7%)			
Eastern Black Sea	229 (3%)^a^	183 (3.9%)^b^	412 (3.3%)			
Northeastern Anatolia	196 (2.6%)^a^	59 (1.2%)^b^	255 (2.1%)			
Eastern Anatolia	282 (3.7%)^a^	236 (5%)^b^	518 (4.2%)			
Southeastern Anatolia	546 (7.1%)^a^	315 (6.6%)^b^	861 (6.9%)			
**Health and Nutrition Characteristics**						
**Presence of chronic disease**	3601 (47%)	2584 (54.5%)	6185 (49.9%)	66.016	**< 0.001** ^**y**^	0.073 ^φ^
**Smoking status**						
None	3533 (46.1%)^a^	2523 (53.2%)^b^	6056 (48.8%)	103.827	**< 0.001** ^**y**^	0.092 [0.079: 0.106] ^V^
Former smoker	1402 (18.3%)^a^	945 (19.9%)^b^	2347 (18.9%)			
Current smoker	2729 (35.6%)^a^	1274 (26.9%)^b^	4003 (32.3%)			
**Physical Activity Level (PAL)**	1.72 (1.1–4.26) / 1.76 ± 0.28	1.75 (1.08–3.84) / 1.77 ± 0.25	1.74 (1.08–4.26) / 1.77 ± 0.27	17228283.500	**< 0.001** ^**x**^	0.087 [0.051: 0.124] ^d^
**Vegetarianism**	49 (0.6%)	27 (0.6%)	76 (0.6%)	0.236	0.627^y^	0.004 ^φ^
**Dieting status**						
Not dieting, satisfied with weight	3200 (41.8%)	2003 (42.3%)	5203 (42%)	23.533	**< 0.001** ^**y**^	0.044 [0.024: 0.068] ^V^
Not dieting, need to lose weight	3226 (42.2%)	1959 (41.4%)	5185 (41.9%)			
Not dieting, need to gain weight	580 (7.6%)^a^	282 (6%)b	862 (7%)			
Currently dieting	643 (8.4)^a^	489 (10.3%)^b^	1132 (9.1%)			
**Food supplement use**	682 (8.9%)	550 (11.6%)	1232 (9.9%)	23.871	**< 0.001** ^**xx**^	0.044 ^φ^
FIES*	-2.287 (-2.29–2.09) / 6177.47 / -1.51 ± 1.32	-2.287 (-2.29–2.09) / 5860.18 / -1.66 ± 1.18	-2.287 (-2.29–2.09) / -1.56 ± 1.27	16465002.000	**< 0.001** ^**x**^	0.089 [0.052: 0.125] ^d^
**Anthropometric and Physiological Characteristics**						
**Body Mass Index (BMI**,** kg/m**^**2**^**)**	27.68 (14.5–93.84) / 28.34 ± 6.14	28.4 (12.11–119.04) / 28.96 ± 6.16	27.95 (12.11–119.04) / 28.58 ± 6.16	16144396.000	**< 0.001** ^**x**^	0.113 [0.076: 0.150] ^d^
**Waist to hip ratio**	0.89 (0.47–1.62) / 0.89 ± 0.09	0.89 (0.51–1.64) / 0.89 ± 0.09	0.89 (0.47–1.64) / 0.89 ± 0.09	16820332.000	0.339^**x**^	0.018 [-0.019: 0.054] ^d^
**Basal Metabolic Rate**	1508.77 (864.72–3011.69) / 1540.77 ± 257.26	1451.68 (621.76–3111.07) / 1492.48 ± 238.56	1483.44 (621.76–3111.07) / 1522.26 ± 251.35	15388865.000	**< 0.001** ^**x**^	0.187 [0.150: 0.224] ^d^
**Total energy expenditure**	2624.6 (1073.77–6951.79) / 2727.33 ± 650.01	2566.48 (888.92–6147.33) / 2658.53 ± 594.05	2600.1 (888.92–6951.79) / 2701 ± 630.04	16292021.000	**< 0.001** ^**x**^	0.099 [0.062: 0.135] ^d^
**Body roundness index (BRI)**	4.75 (0.79–20.31) / 5 ± 2.32	5.09 (0.63–22.11) / 5.31 ± 2.34	4.87 (0.63–22.11) / 5.12 ± 2.33	15579630.500	**< 0.001** ^**x**^	0.145 [0.108: 0.182]^d^

^x^Mann Whitney U Test; values are presented as median (IQR) / mean ± SD for continuous variables and n (%) for categorical variables. ^xx^Fisher’s Exact Test with Monte Carlo correction; ^y^Pearson Chi-Square Test; n (%); ^a−b^Categories sharing the same superscript letter do not differ significantly in post-hoc pairwise comparisons. ^1^:15–18 years, ^2^:19–64 years, ^3^:65 years and more, n (%); ^4^Income level 1: More than adequate to comfortably cover monthly expenses, ^5^Income level 2: Adequate to meet monthly needs without difficulty, ^6^Income level 3: Sufficient for basic needs, but financial strain is present, ^7^Income level 4: Insufficient to meet basic needs, *FIES: Food Insecurity Experience Scale score (continuous), it is a Rasch-calibrated logit score; values may be negative depending on scale centering. ES [95% CI]= Effect Size [95% Confidence Interval]: Cohen’s d (d); ES = Cramer’s V (V) and Phi (φ); 95% CI bootstrap ; n(%)

### Food security psychometrics and associations with fruit, Vegetable, and freshly squeezed juice intake

Rasch modeling of the FIES supported the expected severity gradient and acceptable item fit for retained items; the WHLDAY item was excluded for misfit (outfit = 57.603), yielding a seven-item scale with acceptable reliability for group comparisons (Rasch reliability = 0.68). Category thresholds were estimated on the logit scale (Supplementary Table S2).

According to the adjusted logistic regression analysis, after controlling for age, sex, and income status, individuals with daily fruit and vegetable consumption had lower odds of moderate food insecurity than those without daily fruit and vegetable consumption (OR = 0.745; 95% CI: 0.666–0.834; *p* < 0.001). Similarly, compared with those who never consumed freshly squeezed fruit juice, individuals with occasional consumption had lower odds of moderate food insecurity (OR = 0.875; 95% CI: 0.785–0.975; *p* = 0.016). In contrast, daily freshly squeezed fruit juice consumption was not significantly associated with moderate food insecurity (OR = 0.865; 95% CI: 0.466–1.608; *p* = 0.647). Age was not significantly associated with moderate food insecurity (OR = 0.998; 95% CI: 0.995–1.001; *p* = 0.254). Men had lower odds of moderate food insecurity than women (OR = 0.815; 95% CI: 0.731–0.908; *p* < 0.001). The odds of moderate food insecurity increased markedly as income status worsened (Table 2).

**Table 2:** Adjusted associations of daily fruit and vegetable consumption and freshly squeezed fruit juice consumption with moderate food insecurity

Daily Fruit and Vegetable Consumption (Ref: Non-Daily Consumers)	OR (%95 CI)	*p*
	0.745 (0.666–0.834)	< 0.001
Daily Freshly Squeezed Fruit Juice Consumption (Ref: Never)		
Occasional	0.875(0.785–0.975)	**0.016**
Daily	0.865(0.466–1.608)	0.647
Age	0.998(0.995–1.001)	0.254
Sex (Referans: Women)	0.815(0.731–0.908)	**< 0.001**
Income status (Ref: Income level 1^1^)		
Income level 2^2^	3.168(2.237–4.488)	**< 0.001**
Income level 3^3^	15.868(11.546–21.808)	**< 0.001**
Income level 4^4^	57.462(41.617–79.339)	**< 0.001**
Do not know	15.91(8.676–29.174)	**< 0.001**

OR (%95 CI): Odds Ratio (%95 Confidence Interval), ^1^Income level 1: More than adequate to comfortably cover monthly expenses, ^2^Income level 2: Adequate to meet monthly needs without difficulty, ^3^Income level 3: Sufficient for basic needs, but financial strain is present, ^4^Income level 4: Insufficient to meet basic needs

#### Multivariable associations with intake outcomes

In adjusted logistic models (*n* = 11,716), daily F&V consumption was independently associated with older age (aOR per year 1.013; 95% CI 1.010–1.017; *P* < 0.001) and female sex (1.577; 1.44–1.727; *P* < 0.001). Using married participants as the reference group, lower odds of daily F&V consumption were observed among those who were never married (aOR 0.792; 95% CI 0.694–0.904; *P* = 0.001), widowed (aOR 0.736; 95% CI 0.632–0.858; *P* < 0.001), and divorced (aOR 0.724; 95% CI 0.584–0.898; *P* = 0.003). Education showed a mixed pattern relative to primary school: illiteracy was associated with lower odds (0.717; 0.612–0.839; *P* < 0.001), whereas some mid-level categories had higher odds (e.g., elementary school 1.324; 1.012–1.734; *P* = 0.041; secondary school 1.254; 1.010–1.558; *P* = 0.041); tertiary was not different from primary (0.999; 0.879–1.136; *P* = 0.988). Food insecurity was inversely associated with daily F&V intake (FIES aOR per unit 0.895; 0.863–0.928; *P* < 0.001). Food supplement use (1.164; 1.023–1.325; *P* = 0.021) and higher physical activity (PAL 1.459; 1.250–1.703; *P* < 0.001) were positively associated. BMI and BRI were not associated after adjustment (both *P* > 0.30). Regional differences persisted versus the Aegean: higher odds in the Mediterranean (1.220; 1.066–1.397; *P* = 0.004) and Eastern Anatolia (1.437; 1.168–1.767; *P* = 0.001), and lower odds in Central Anatolia (0.572; 0.469–0.697; *P* < 0.001), Northeastern Anatolia (0.476; 0.345–0.658; *P* < 0.001), Western Anatolia (0.614; 0.509–0.739; *P* < 0.001), Eastern Anatolia (0.638; 0.547–0.743; *P* < 0.001), and Istanbul (0.865; 0.754–0.992; *P* = 0.038); other regions were not different. Multivariable regression models were adjusted by including all covariates shown in Table 3 simultaneously (sex, age, marital status, education, income status, NUTS regions, physician-diagnosed chronic disease, dieting status, smoking status, dietary supplement use, physical activity level, BMI, and FIES score). Analyses were conducted using complete-case data for participants with non-missing values on these covariates.

**Table 3:** Binary logistic regression of factors associated with daily fruit and vegetable consumption (n:11,716)

	Univariate				Multiple			
	OR (%95 CI)		*P*		OR (%95 CI)		*P*	
Sex (Ref: Men)								
Women	1.545 (1.435–1.662)		**< 0.001**		1.577 (1.44–1.727)		**< 0.001**	
Marital status (Ref: Married)								
Single	0.616 (0.556–0.682)		**< 0.001**		0.792 (0.694–0.904)		**0.001**	
Widowed	1.059 (0.935–1.199)		0.366		0.736 (0.632–0.858)		**< 0.001**	
Divorced	0.75 (0.613–0.918)		**0.005**		0.724 (0.584–0.898)		**0.003**	
Separated	1.006 (0.577–1.754)		0.983		1.043 (0.584–1.866)		0.886	
Education status (Ref: Primary school)								
Illiterate	0.885 (0.775–1.009)		0.068		0.717 (0.612–0.839)		**< 0.001**	
Literate	1.034 (0.859–1.245)		0.724		0.921 (0.754–1.126)		0.424	
Primary school graduate	1.033 (0.804–1.328)		0.797		1.324 (1.012–1.734)		**0.041**	
Middle school	0.746 (0.653–0.852)		**< 0.001**		0.964 (0.834–1.116)		0.626	
Middle school graduate	0.848 (0.699–1.029)		0.095		1.254 (1.01–1.558)		**0.041**	
High school equivalent	0.724 (0.653–0.803)		**< 0.001**		0.981 (0.869–1.108)		0.759	
University	0.807 (0.724–0.898)		**< 0.001**		0.999 (0.879–1.136)		0.988	
Presence of disease (Ref: No)								
Yes	1.351 (1.256–1.453)		**< 0.001**		1.076 (0.985–1.176)		0.105	
Dieting status (Ref: Yes)								
Not dieting, satisfied with weight	0.823 (0.723–0.938)		**0.003**		0.903 (0.779–1.046)		0.174	
Not dieting, need to lose weight	0.798 (0.701–0.91)		**0.001**		0.78 (0.68–0.895)		**< 0.001**	
Not dieting, need to gain weight	0.639 (0.532–0.769)		**< 0.001**		0.882 (0.712–1.092)		0.250	
Food supplement use (Ref: No)								
Yes	1.343 (1.193–1.512)		**< 0.001**		1.164 (1.023–1.325)		**0.021**	
NUTS Regions (Ref: Aegean)								
Istanbul	0.817 (0.718–0.931)		**0.002**		0.865 (0.754–0.992)		**0.038**	
Western Marmara		0.615 (0.514–0.734)		**< 0.001**		0.614 (0.509–0.739)		**< 0.001**
Eastern Marmara		0.638 (0.551–0.738)		**< 0.001**		0.638 (0.547–0.743)		**< 0.001**
Western Anatolia		0.903 (0.783–1.041)		0.159		0.912 (0.786–1.058)		0.222
Mediterranean		1.162 (1.022–1.321)		**0.022**		1.22 (1.066–1.397)		**0.004**
Central Anatolia		0.572 (0.473–0.692)		**< 0.001**		0.572 (0.469–0.697)		**< 0.001**
Western Black Sea		1.003 (0.853–1.18)		0.968		1.015 (0.855–1.206)		0.861
Eastern Black Sea		1.127 (0.911–1.394)		0.269		1.122 (0.896–1.407)		0.316
Northeastern Anatolia		0.425 (0.313–0.575)		**< 0.001**		0.476 (0.345–0.658)		**< 0.001**
Eastern Anatolia		1.18 (0.973–1.432)		0.092		1.437 (1.168–1.767)		**0.001**
Southeastern Anatolia		0.814 (0.691–0.958)		**0.013**		1.048 (0.878–1.25)		0.605
Income status (Ref: Income level 1^1^)								
Income level 2^2^		0.927 (0.834–1.03)		0.159		0.915 (0.818–1.022)		0.117
Income level 3^3^		0.958 (0.868–1.057)		0.394		0.959 (0.859–1.07)		0.453
Income level 4^4^		0.831 (0.737–0.938)		**0.003**		0.966 (0.835–1.117)		0.641
Do not know		0.734 (0.481–1.12)		0.151		0.8 (0.497–1.287)		0.358
Smoking Status (Ref: No)								
Former smoker		1.53 (1.407–1.664)		**< 0.001**		1.291 (1.172–1.423)		**< 0.001**
Current smoker		1.444 (1.299–1.605)		**< 0.001**		1.277 (1.138–1.433)		**< 0.001**
Age (years)		1.012 (1.01–1.014)		**< 0.001**		1.013 (1.01–1.017)		**< 0.001**
FIES*		0.91 (0.883–0.937)		**< 0.001**		0.895 (0.863–0.928)		**< 0.001**
Body Mass Index (BMI, kg/m^2^)		1.016 (1.010–1.022)		**< 0.001**		0.993 (0.98–1.006)		0.306
Body roundness index (BRI)		1.058 (1.041–1.076)		**< 0.001**		1.019 (0.982–1.059)		0.320
Physical Activity Level (PAL)		1.184 (1.035–1.354)		**0.014**		1.459 (1.25–1.703)		**< 0.001**

OR (%95 CI): Odds Ratio (%95 Confidence Interval), ^1^Income level 1: More than adequate to comfortably cover monthly expenses, ^2^Income level 2: Adequate to meet monthly needs without difficulty, ^3^Income level 3: Sufficient for basic needs, but financial strain is present, ^4^Income level 4: Insufficient to meet basic needs, *FIES: Food Insecurity Experience Scale score (continuous)

For freshly squeezed fruit juice, multinomial logistic models with “never” as the reference category showed that higher education (relative to primary; e.g., high school aOR 1.805, 95% CI 1.605–2.029; higher education aOR 2.381, 95% CI 2.102–2.697; both *P* < 0.001) and dietary supplement use (aOR 1.226, 95% CI 1.078–1.395; *P* = 0.002) were associated with higher odds of being an occasional consumer versus a never consumer. Higher food insecurity was associated with lower odds of being an occasional consumer versus a never consumer (aOR per unit 0.955, 95% CI 0.926–0.986; *P* = 0.004), and both former (aOR 0.864, 95% CI 0.787–0.948; *P* = 0.002) and current smoking (aOR 0.885, 95% CI 0.791–0.989; *P* = 0.032) were associated with lower odds of occasional consumption versus never.

In contrast, daily consumption versus never (rare overall) was associated with dietary supplement use (aOR 1.750, 95% CI 1.016–3.014; *P* = 0.044) and was less likely among those reporting a need to lose weight (aOR 0.423, 95% CI 0.236–0.758; *P* = 0.004); other covariates were not statistically significant (all *P* ≥ 0.051) (Table 4). After mutual adjustment, the pattern simplified: the education gradient, supplement use, and inverse associations with food insecurity and smoking remained for occasional versus never intake, whereas for daily versus never intake only supplement use remained positively associated and weight-loss intention remained inversely associated **(**Table 4**)**.

**Table 4:** Multinomial logistic regression of factors associated with fruit juice consumption (*n* = 11,691)

	Other Consumption^a^		Daily Consumption	
	OR (%95 GA)	*P*	OR (%95 GA)	*P*
Sex (Ref: Men)				
Women	1.065 (0.975–1.163)	0.161	1.473 (0.951–2.283)	0.083
Marital status (Ref: Married)				
Single	0.894 (0.789–1.013)	0.080	0.723 (0.396–1.321)	0.292
Widowed	0.986 (0.844–1.151)	0.855	1.257 (0.546–2.897)	0.591
Divorced	0.886 (0.721–1.09)	0.252	1.153 (0.454–2.933)	0.764
Separated	1.073 (0.599–1.922)	0.812	2.724 (0.353–21.006)	0.336
Education status (Ref: Primary school)				
Illiterate	0.694 (0.591–0.814)	**< 0.001**	0.431 (0.145–1.277)	0.129
Literate	0.913 (0.746–1.116)	0.374	1.018 (0.355–2.925)	0.973
Primary school graduate	1.488 (1.145–1.935)	**0.003**	1.49 (0.442–5.023)	0.520
Middle school	1.408 (1.226–1.617)	**< 0.001**	0.757 (0.337–1.697)	0.499
Middle school graduate	1.318 (1.07–1.624)	**0.010**	1.394 (0.538–3.614)	0.494
High school equivalent	1.805 (1.605–2.029)	**< 0.001**	1.748 (0.998–3.061)	0.051
University	2.381 (2.102–2.697)	**< 0.001**	1.563 (0.841–2.905)	0.158
Presence of disease (Ref: No)				
Yes	0.94 (0.862–1.024)	0.158	0.772 (0.497–1.198)	0.249
Dieting status (Ref: Yes)				
Not dieting, satisfied with weight	0.748 (0.646–0.867)	**< 0.001**	0.556 (0.3–1.031)	0.062
Not dieting, need to lose weight	0.88 (0.767–1.01)	0.069	0.423 (0.236–0.758)	**0.004**
Not dieting, need to gain weight	0.629 (0.511–0.775)	**< 0.001**	0.719 (0.303–1.708)	0.455
Food supplement use (Ref: No)				
Yes	1.226 (1.078–1.395)	**0.002**	1.75 (1.016–3.014)	**0.044**
Smoking Status (Ref: No)				
Former smoker	0.864 (0.787–0.948)	**0.002**	0.909 (0.577–1.43)	0.679
Current smoker	0.885 (0.791–0.989)	**0.032**	0.906 (0.506–1.621)	0.739
Age (years)	1.003 (0.999–1.007)	0.097	0.99 (0.971–1.009)	0.301
FIES*	0.955 (0.926–0.986)	**0.004**	0.867 (0.726–1.036)	0.116
Body Mass Index (BMI, kg/m^2^)	1.013 (0.999–1.026)	0.068	0.974 (0.907–1.046)	0.469
Body roundness index (BRI)	0.925 (0.89–0.961)	**< 0.001**	1.044 (0.859–1.269)	0.667
Physical Activity Level (PAL)	1.134 (0.976–1.317)	0.100	1.841 (0.925–3.667)	0.082

OR (%95 CI): Odds Ratio (%95 Confidence Interval), *Includes Occasional and Never, ^a^FIES: Food Insecurity Experience Scale score (continuous) Multinomial logistic regression with “never” as the reference category; estimates compare (i) occasional vs. never and (ii) daily vs. never

Together, these models indicate that daily F&V intake is higher among older adults, women, those with greater physical activity and supplement use, and those with lower food insecurity, with substantial regional heterogeneity. Freshly squeezed juice intake, particularly occasional consumption, was more common with higher education and supplement use, and less common at higher levels of food insecurity.

## Discussion

This analysis of nationally representative TNHS-2017 data indicates that daily fruit and vegetable (F&V) consumption in Türkiye varies meaningfully by age, sex, food security, dieting status, and region. Older age and female sex were associated with higher odds of daily intake, food insecurity showed an inverse association with daily intake, and marked geographic heterogeneity was evident across NUTS1 regions. Associations for freshly squeezed juice differed from F&V, with occasional intake linked to higher education and supplement use and lower at higher levels of food insecurity.

Age and sex differences align with prior population studies in which diet quality indices (e.g., HEI-like measures) tend to be higher among older adults and women [26, 27]. Older adults may prioritize cardiometabolic risk reduction and adhere more closely to dietary guidance, while women often report greater engagement with health information and preventive behaviors [28, 29]. Similar patterns have been observed elsewhere: a rapid review in sub-Saharan Africa found that most studies reported increased F&V consumption with age, although results varied across contexts [30], and a large Chinese survey showed higher fruit and vegetable intake among older adults, women, and higher-income groups, with current smokers consuming less fruit [31]. Together, these findings suggest that younger adults and men may warrant consideration in tailored demand-side strategies, alongside other criteria relevant to public health priority setting.

Food insecurity was consistently related to lower daily F&V intake in both descriptive and multivariable analyses, reinforcing the role of economic access in shaping everyday dietary choices. The FIES was calibrated with a Rasch model and the “WHLDAY” item removed for misfit; no respondents reached the severe category, limiting variability at the highest insecurity level. Even within this restricted range, higher FIES scores were associated with lower odds of daily F&V intake and with greater likelihood of non-consumption of freshly squeezed juice. Similar associations have been documented in high-income settings: studies in Europe and the United States consistently show that adults in food-insecure households consume fewer fruits and vegetables than their food-secure counterparts [32–34]. These findings support interventions that address affordability (price supports, vouchers), availability (retail access in lower-income neighborhoods), and stability (mitigating price volatility). The lack of an independent association for household economic situation after adjustment may reflect the coarse measurement of income categories and overlap with FIES, which more directly captures experienced affordability constraints relevant to daily produce consumption.

Dieting status showed a nuanced relationship with outcomes. Individuals reporting a need to lose weight were less likely to consume freshly squeezed juice daily, while occasional juice intake tracked with supplement use and higher education. In univariate models, BMI and BRI correlated with daily fruit and vegetable consumption; however, these associations attenuated and became non-significant after full adjustment, indicating that body size indicators are not independent predictors of fruit and vegetable intake. Rather, consumption patterns reflect sociodemographic and behavioral determinants, including sex, age, education and income, region, smoking, and physical activity, consistent with prior population studies. Notably, active weight-management (dieting) remained associated with higher F&V intake, aligning with evidence that people attempting weight loss commonly increase F&V. Together with trials showing that increasing F&V without energy reduction has little effect on body weight [35], our findings suggest policies should prioritize equity, access, and behavior change (e.g., smoking cessation, PA, diet counseling) over weight status per se. These relationships are summarized in **Fig. 2**, which illustrates the independent sociodemographic, regional, lifestyle, and dietary factors associated with daily fruit and vegetable intake, as well as the absence of an independent association for BMI/BRI after adjustment. Similarly, evidence from U.S. adolescents shows that those intending to lose weight were more likely to meet fruit and vegetable recommendations, underscoring that weight-control intentions, rather than body size alone, may drive higher consumption [36]. Together with the unadjusted higher BMI/BRI in daily F&V consumers but null associations after adjustment, these results are compatible with reverse causation (health-motivated changes among those with higher adiposity) and with substitution dynamics in which juice is perceived as a “healthy convenience” among health-attentive groups. Public messaging may need to emphasize whole-fruit preference and appropriate use of 100% juice within recommended limits.

**Fig. 1:**
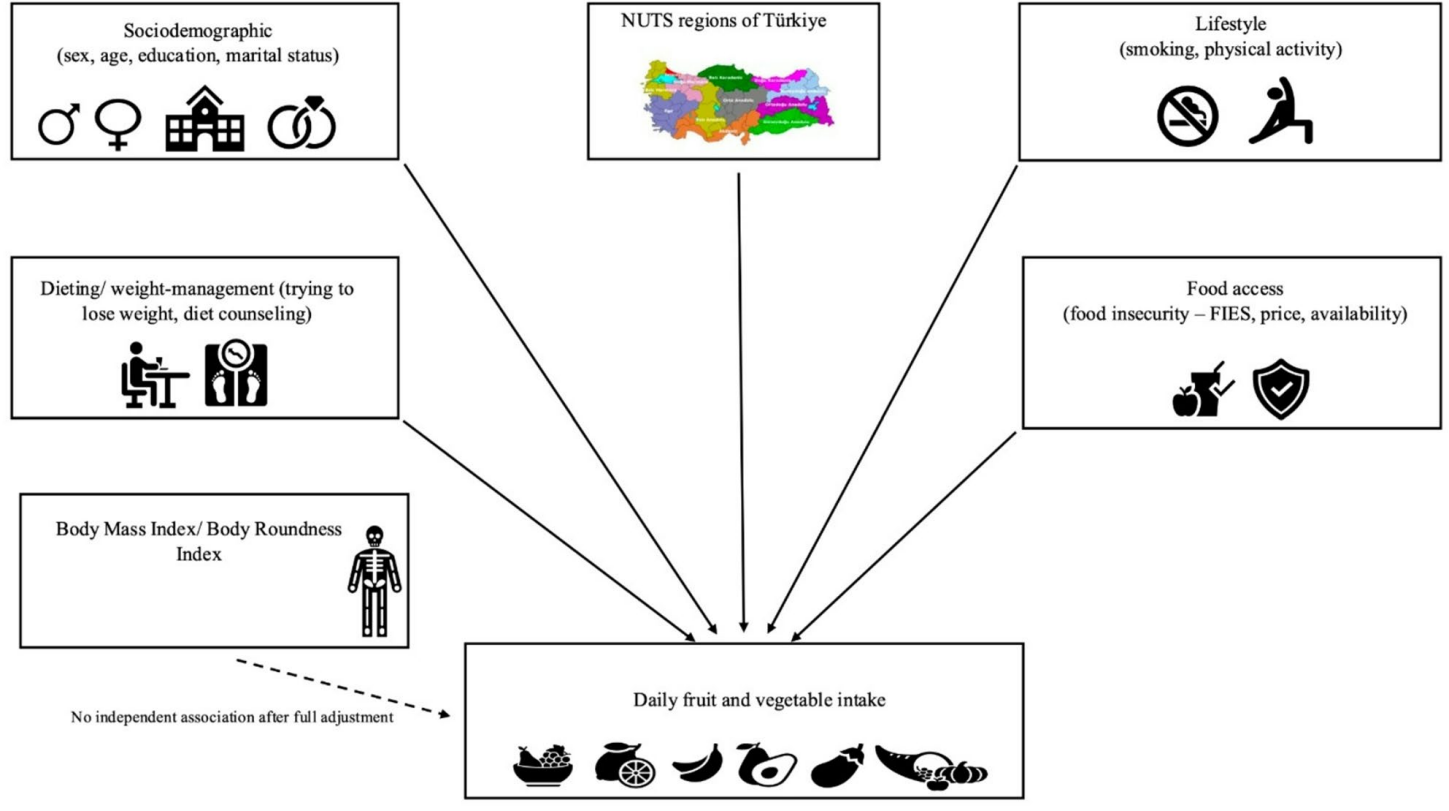
Factors associated with daily fruit and vegetable intake in the Türkiye Nutrition and Health Survey.* * Solid arrows represent sociodemographic, regional, lifestyle, dietary, and food access factors that were independently associated with daily fruit and vegetable intake. The dashed arrow indicates that Body Mass Index and Body Roundness Index were not independently associated after full multivariable adjustment

Regional disparities were pronounced even after adjustment. Compared with the Aegean, odds of daily F&V intake were lower in regions such as Central and higher in Mediterranean and Middle East Anatolia. These contrasts likely reflect differences in agro-ecology, prices and supply chains, cultural foodways, urban–rural retail structures, and local economic conditions. Consistent with our findings, the most recent Global Burden of Disease analysis reported widening gaps in F&V consumption between high- and low-Sociodemographic Index countries from 1990 to 2021, with declines in F&V-related mortality occurring more rapidly in wealthier regions, suggesting a growing nutrition divide [7]. Beyond national averages, subnational and regional disparities in F&V intake are often profound, with certain areas lagging behind despite similar policy frameworks [27, 37]. National dietary surveys across multiple countries confirm a socioeconomic gradient in F&V intake: lower-income, less-educated, and marginalized populations are consistently less likely to meet recommended intake levels, exacerbating the risk of diet-related chronic disease. Thus, F&V consumption represents not only a health concern but also an issue of social justice, reflecting broader social determinants of diet quality. The within-country spread underscores the limitation of national averages and supports region-tailored strategies—such as improving cold-chain distribution inland, leveraging regional cuisines that traditionally feature vegetables, and aligning municipal food policies (procurement for schools, marketplaces) with affordability goals.

Contextualizing these results within recent monitoring trends, Türkiye has experienced a decline in average F&V intake despite high agricultural output. As of 2023, Türkiye ranked fourth worldwide in fruit and vegetable production, with ~ 26 million tons of fruit and ~ 32 million tons of vegetables harvested annually [38]. FAO data from 2021 indicate that per capita fruit and vegetable availability in Türkiye far exceeds that of the European Union—382 g/day of fruit and 681 g/day of vegetables, compared to 307 g/day and 414 g/day in the EU, respectively [39].

On the surface, these aggregate figures suggest a relatively favorable dietary profile. Yet nutritional monitoring data paints a different picture. According to the Türkiye Health Surveys (2014–2022) of TurkStat, the proportion of adults consuming zero portions of fruits and vegetables on a given day increased markedly, from 33.6% in 2014 to 53.3% in 2022. The increase was observed in both sexes (men: 35.7% to 56.1%; women: 31.5% to 50.6%), with men showing a consistently higher prevalence at both time points [40]. Meanwhile, the proportion of individuals consuming the WHO-recommended ≥ 5 portions per day declined from 3.1% in 2014 to just 2.3% in 2022, indicating worsening alignment with dietary guidelines. Also, those consuming the moderate range of 1–4 portions/day dropped from 63.4% to 44.4% within eight years [40]. These changes suggest that not only is the population failing to improve, but even previously moderate consumers are shifting toward zero intake, a worrying signal for diet quality and chronic disease prevention. These figures underscore the disconnect between agricultural abundance and population-level dietary adequacy, raising urgent questions about access, affordability, and structural barriers to consumption, even in food-producing nations. The present findings suggest plausible mechanisms: economic barriers captured by FIES, demographic segments with lower intake (younger adults, men), and region-specific constraints. Future research could extend this work by linking TNHS consumption data with region-level fruit and vegetable production and other food-system indicators, such as prices, market access, and supply-chain characteristics, to better understand how agricultural capacity translates into dietary intake. Interventions that couple national guidance (TÜBER 2022) with targeted affordability measures and region-appropriate implementation may be necessary to reverse the downward trajectory.

### Strengths and Limitations

The study draws on a large, nationally representative sample selected via multistage stratified cluster sampling; data collection was standardized by trained dietitians; anthropometrics were measured rather than self-reported; and food insecurity was calibrated with a Rasch model, supporting group-level comparisons. Data collection spanned February–August, covering late winter through summer and thereby capturing a broad range of seasonal dietary patterns in Türkiye’s diverse climate. The convergence of findings across descriptive and multivariable analyses, together with sufficient power to estimate subnational differences, further supports internal validity. However, several limitations warrant consideration. First, the cross-sectional design precludes causal inference, and the temporal direction of observed associations cannot be established (e.g., individuals with chronic disease or higher adiposity may increase fruit and vegetable intake following diagnosis or health advice). In future studies specifically designed for causal inference, directed acyclic graphs may help define exposure-specific adjustment sets and distinguish more clearly between total and direct effects. Second, FFQ-based frequency data classify daily vs. non-daily intake without portion quantification and remain subject to recall and social-desirability bias. Moreover, daily fruit and vegetable consumption was a common outcome in this study; accordingly, odds ratios from logistic regression may overstate associations relative to prevalence ratios and should be interpreted with caution. In addition, because the primary outcomes were derived from FFQ-based frequency measures rather than energy intake estimates, under-reporting was not formally assessed using energy intake–based methods; therefore, possible misreporting of usual intake cannot be excluded. Third, the upper tail of the food insecurity (FIES) distribution was constrained, as no respondents met the threshold for severe food insecurity, and one item (WHLDAY) was removed because of substantial misfit prior to calibration. Although the Rasch reliability coefficient of 0.68 was acceptable for the final 7-item scale according to FAO guidance, the restricted variability at the more severe end of the distribution may have attenuated observed gradients across food insecurity levels.

Finally, residual confounding by unmeasured food-environment factors (such as regional food prices and retail access) is possible, since these factors were not explicitly measured in TNHS-2017. TNHS-2017 is the most recent nationally representative dietary dataset available for Türkiye; however, the survey was conducted nearly a decade ago. Since 2017, macroeconomic conditions, food price inflation, and food-system dynamics may have altered fruit-and-vegetable affordability and consumption patterns; therefore, our estimates should be interpreted as a national benchmark rather than a precise representation of current intake. In addition, the TNHS-2017 FFQ items used in this analysis capture frequency categories rather than gram or portion quantities and aggregate fruit and vegetables at the “total” level; therefore, we could not quantify adherence to the WHO ≥ 400 g/day recommendation or model more granular intake patterns beyond the frequency-based definitions reported here.

### Implications

This study highlights practical actions to increase fruit and vegetable intake in Türkiye. Given that non-daily consumption is common across the population and monitoring data suggest worsening trends, policies should combine universal approaches (e.g., strengthening implementation of TÜBER guidance, improving affordability and availability, and public communication promoting whole-produce intake) with targeted strategies for groups and settings with consistently lower intake. In the present analyses, younger adults, men, and individuals with higher food insecurity scores showed lower daily consumption, suggesting that additional demand-creation and access supports may be needed in these groups and in lower-intake regions. However, these subgroup differences should not be interpreted in isolation when setting intervention priorities, which should also consider broader health risk profiles, feasibility, cost-effectiveness, and the likely impact of specific interventions. Communication should emphasize whole fruits and vegetables while providing clear guidance on the limited role of 100% fruit juice, which may complement but should not replace whole fruits. Integrating brief FIES-based screening into primary care and community nutrition services could help identify vulnerable households for targeted support. Interventions should be evaluated using longitudinal or quasi-experimental designs that incorporate food environment and price data. Strengthening consumer trust in produce safety is also essential, given concerns about pesticide residues and reports of non-compliant products [41, 42]. Coordinated efforts to improve affordability, access, education, and trust are likely to yield the greatest public health gains.

## Conclusion

In a nationally representative sample of individuals aged ≥ 15 years, daily fruit and vegetable intake was higher among women and older adults, inversely associated with food insecurity, and varied markedly across regions. Physical activity and supplement use were positively associated with intake, while anthropometric indicators showed little independent effect. Freshly squeezed fruit juice followed a distinct pattern: occasional use was more common among higher-educated and supplement-using groups and less common among the food-insecure; yet daily use was rare. Reversing the decline in fruit and vegetable consumption in Türkiye will likely require policies that improve affordability and access, strengthen regional food systems, and pair national guidance with demand-creation strategies.

## Supplementary Information

Below is the link to the electronic supplementary material.

Supplementary Material 1

## Data Availability

This study used data obtained from the Türkiye Ministry of Health. The dataset can be accessed from the Ministry upon reasonable request and completion of the procedures described in the article.
