# Supplementary material for: Agricultural abundance, dietary shortfall: national determinants of daily fruit and vegetable intake in Türkiye National Nutrition and Health Survey
Source: J Health Popul Nutr. 2026 Apr 13;45:136. doi: 10.1186/s41043-026-01310-0 (PMC13185294; doi:10.1186/s41043-026-01310-0)
Supplement: Supplementary file 1 — Supplementary Material 1 [file 41043_2026_1310_MOESM1_ESM.docx]

**Supplementary Tables for** **Agricultural abundance, dietary shortfall: National determinants of daily fruit and vegetable intake in Türkiye National Nutrition and Health Survey**

**Supplementary Table 1a.** Comparison of categorical sociodemographic, health, lifestyle, and food insecurity-related characteristics by weekly fruit and vegetable consumption status

|  | **Those consuming no more than once per week** | **Those consuming more than once per week** | **Test Statistic** | **p** | **ES [95% CI]** |
| --- | --- | --- | --- | --- | --- |
| **Age group** |  |  |  |  |  |
| Adolescent^1^ | 114 (6.3) ^a^ | 412 (3.9) ^b^ | 55.110 | **<0.001^x^** | 0.067 [0.046: 0.082] ^v^ |
| Adult^2^ | 1451 (80.6) ^a^ | 8169 (77) ^b^ |  |  |  |
| Older Adult^3^ | 235 (13.1) ^a^ | 2025 (19.1) ^b^ |  |  |  |
| **Education status** |  |  |  |  |  |
| Illiterate | 161 (8.9) ^a^ | 1037 (9.8) ^a^ | 49.729 | **<0.001^x^** | 0.063 [0.044: 0.081] ^v^ |
| Literate | 65 (3.6) ^a^ | 448 (4.2) ^a^ |  |  |  |
| Primary school | 498 (27.7) ^a^ | 3624 (34.2) ^b^ |  |  |  |
| Primary school graduate | 38 (2.1) ^a^ | 228 (2.2) ^a^ |  |  |  |
| Middle school | 218 (12.1) ^a^ | 1010 (9.5) ^b^ |  |  |  |
| Middle school graduate | 79 (4.4) ^a^ | 406 (3.8) ^a^ |  |  |  |
| High school equivalent | 428 (23.8) ^a^ | 2041 (19.2) ^b^ |  |  |  |
| University | 313 (17.4) ^a^ | 1812 (17.1) ^a^ |  |  |  |
| **Sex** |  |  |  |  |  |
| Women | 895 (49.7) | 5688 (53.6) | 9.435 | **0.002^x^** | 0.028 ^φ^ |
| Men | 905 (50.3) | 4918 (46.4) |  |  |  |
| **Marital status** |  |  |  |  |  |
| Never married | 457 (25.4) ^a^ | 1710 (16.1) ^b^ | 88.095 | **<0.001^y^** | 0.088 [0.068: 0.110] ^v^ |
| Married | 1123 (62.4) ^a^ | 7453 (70.3) ^b^ |  |  |  |
| Widowed | 143 (7.9) ^a^ | 1027 (9.7) ^b^ |  |  |  |
| Divorced | 70 (3.9) ^a^ | 371 (3.5) ^a^ |  |  |  |
| Separated | 7 (0.4) ^a^ | 45 (0.4) ^a^ |  |  |  |
| **Income status** |  |  |  |  |  |
| Income level 1^4^ | 400 (22.2) ^a^ | 2235 (21.1) ^a^ | 10.389 | **0.033^y^** | 0.029 [0.015: 0.057] ^v^ |
| Income level 2^5^ | 436 (24.2) ^a^ | 2800 (26.4) ^a^ |  |  |  |
| Income level 3^6^ | 626 (34.8) ^a^ | 3829 (36.1) ^a^ |  |  |  |
| Income level 4^7^ | 325 (18.1) ^a^ | 1654 (15.6) ^b^ |  |  |  |
| Do not know | 13 (0.7) ^a^ | 88 (0.8) ^a^ |  |  |  |
| **NUTS regions** |  |  |  |  |  |
| Istanbul | 278 (15.4) ^a^ | 1469 (13.9) ^a^ | 83.259 | **<0.001^x^** | 0.082 [0.066: 0.107] ^v^ |
| Western Marmara | 119 (6.6) ^a^ | 629 (5.9) ^a^ |  |  |  |
| Aegean | 276 (15.3) ^a^ | 1879 (17.7) ^b^ |  |  |  |
| Eastern Marmara | 257 (14.3) ^a^ | 1024 (9.7) ^b^ |  |  |  |
| Western Anatolia | 189 (10.5) ^a^ | 1064 (10) ^a^ |  |  |  |
| Mediterranean | 163 (9.1) ^a^ | 1535 (14.5) ^b^ |  |  |  |
| Central Anatolia | 109 (6.1) ^a^ | 539 (5.1) ^a^ |  |  |  |
| Western Black Sea | 108 (6) ^a^ | 722 (6.8) ^a^ |  |  |  |
| Eastern Black Sea | 50 (2.8) ^a^ | 362 (3.4) ^a^ |  |  |  |
| Northeastern Anatolia | 46 (2.6) ^a^ | 209 (2) ^a^ |  |  |  |
| Eastern Anatolia | 74 (4.1) ^a^ | 444 (4.2) ^a^ |  |  |  |
| Southeastern Anatolia | 131 (7.3) ^a^ | 730 (6.9) ^a^ |  |  |  |
| ‎**Presence of chronic disease** | 782 (43.4) | 5403 (50.9) | 34.610 | **<0.001^x^** | 0.053 ^φ^ |
| **Smoking status** |  |  |  |  |  |
| None | 786 (43.7) ^a^ | 5270 (49.7) ^b^ | 84.466 | **<0.001^x^** | 0.083 [0.067: 0.107] ^v^ |
| Former smoker | 268 (14.9) ^a^ | 2079 (19.6) ^b^ |  |  |  |
| Current smoker | 746 (41.4) ^a^ | 3257 (30.7) ^b^ |  |  |  |
| ‎**Vegetarianism** | 15 (0.8) | 61 (0.6) | 1.287 | 0.257^z^ | 0.012 ^φ^ |
| **Dieting status** |  |  |  |  |  |
| Not dieting, satisfied with weight | 750 (41.7) ^a^ | 4453 (42.1) ^a^ | 13.823 | **0.003^x^** | 0.033 [0.010: 0.066]^v^ |
| Not dieting, need to lose weight | 735 (40.9) ^a^ | 4450 (42) ^a^ |  |  |  |
| Not dieting, need to gain weight | 161 (9) ^a^ | 701 (6.6) ^b^ |  |  |  |
| Currently dieting | 151 (8.4) ^a^ | 981 (9.3) ^a^ |  |  |  |
| **Food supplement use** | 135 (7.5) | 1097 (10.3) | 13.908 | **<0.001^x^** | 0.034^φ^ |

^x^ Pearson’s chi-squared test; ^y^ Fisher’s exact test with Monte Carlo simulation; ^z^ Pearson’s chi-squared test with Yates’s continuity correction; ^a-b^ no significant difference between groups sharing the same letter; multiple comparisons were examined using the pairwise Z test; ES = effect size; V = Cramér’s V; φ = Phi coefficient; 95% CI was calculated using the bootstrap method; values are presented as n (%); ^1^:15-18 years, ^2^:19-64 years, ^3^:65 years and more; ^4^Income level 1: More than adequate to comfortably cover monthly expenses, ^5^Income level 2: Adequate to meet monthly needs without difficulty, ^6^Income level 3: Sufficient for basic needs, but financial strain is present, ^7^Income level 4: Insufficient to meet basic needs,

**Supplementary Table 1b.** Comparison of quantitative variables according to weekly fruit and vegetable consumption status

|  | **Those consuming no more than once per week** | **Those consuming more than once per week** | **Test Statistic** | **p** | **ES [95% CI]** |
| --- | --- | --- | --- | --- | --- |
| **AGE** | 40 (15: 105) / 42.48 ± 17.68 | 46 (16: 100) / 47.64 ± 17.62 | -12.030 | **<0.001**^x^ | 0.217 [0.167: 0.267] |
| **FIES scale score** | -2.29 (-2.29: 2.09) / -1.42 ± 1.4 | -2.29 (-2.29: 2.09) / -1.59 ± 1.24 | -3.234 | **0.001**^x^ | 0.059 [0.008: 0.110] |
| **Body Mass Index** | 26.94 (15.76: 68.23) / 27.77 ± 6.21 | 28.12 (12.11: 119.04) / 28.71 ± 6.14 | -7.236 | **<0.001**^x^ | 0.132 [0.081: 0.183] |
| **Waist to hip ratio** | 0.88 (0.65: 1.29) / 0.88 ± 0.09 | 0.89 (0.47: 1.64) / 0.89 ± 0.09 | -5.397 | **<0.001**^x^ | 0.099 [0.048: 0.150] |
| **Basal Metabolic Rate** | 1500.54 (897.6: 2920.67) / 1540.42 ± 268.87 | 1480.61 (621.76: 3111.07) / 1519.22 ± 248.16 | -2.512 | **0.012**^x^ | 0.046 [-0.005: 0.096] |
| **Total energy expenditure** | 2641.17 (1152.26: 6274.19) / 2728.41 ± 673.3 | 2595.65 (888.92: 6951.79) / 2696.37 ± 622.35 | -1.339 | 0.181^x^ | 0.024 [-0.026: 0.075] |
| **Body roundness index (BRI)** | 4.47 (-1.3: 17.07) / 4.72 ± 2.22 | 4.91 (-1.3: 22.11) / 5.19 ± 2.34 | -8.266 | **<0.001**^x^ | 0.151 [0.100: 0.201] |
| **Physical Activity Level (PAL)** | 1.72 (1.11: 4.26) / 1.77 ± 0.3 | 1.74 (1.08: 3.84) / 1.77 ± 0.26 | -2.188 | **0.029**^x^ | 0.039 [-0.011: 0.089] |

^x^ Mann-Whitney U test; values are presented as median (minimum: maximum) / mean ± standard deviation; ES [95% CI] = effect size [95% confidence interval]: Cohen’s d.

‎

**Supplementary Table 2.** Food insecurity items and item fit statistics after the WHLDAY* item is removed

| Label | Affirmative Responses (n. %) | Severity±SE^1^ | Infit^2^ | Outfit^3^ | Probability of moderate or severe FI by raw score | Probability of severe FI by raw score |
| --- | --- | --- | --- | --- | --- | --- |
| Worried^a^ | 2324 (21.08%) | -1220±0.044 | 1229 | 1471 | 0.031 | 0.000 |
| Healthy^b^ | 2227 (20.2%) | -1070±0.044 | 0.907 | 1040 | 0.110 | 0.000 |
| Fewfood^c^ | 2208 (20.03%) | -1041±0.044 | 0.903 | 1005 | 0.343 | 0.000 |
| Skipped^d^ | 1008 (9.15%) | 0.885±0.047 | 0.904 | 0.935 | 0.673 | 0.001 |
| AteLess^e^ | 1459 (13.24%) | 0.119±0.044 | 0.878 | 0.855 | 0.898 | 0.015 |
| RunOut^f^ | 1360 (12.34%) | 0.279±0.044 | 1036 | 1093 | 0.974 | 0.208 |
| Hungry^g^ | 437 (3.96%) | 2047±0.057 | 1030 | 1103 | 0.991 | 0.583 |
| Threshold Value^4^ |  |  |  |  | -0.070 | 3.180 |

*Rasch reliability was 0.68*

*Wholeday item has been removed because its outfit value is 57.603*

^a^ You were worried you would not have enough food to eat because of a lack of money or other resources?

^b^ You were unable to eat healthy and nutritious food because of a lack of money or other resources?

^c^ You ate only a few kinds of foods because of a lack of money or other resources?

^d^ You had to skip a meal because there was not enough money or other resources to get food?

^e^ You ate less than you thought you should because of a lack of money or other resources?

^f^ Your household ran out of food because of a lack of money or other resources?

^g^ You were hungry but did not eat because there was not enough money or other resources for food?

*You went without eating for a whole day because of a lack of money or other resources?

^1:^ Severity parameter of the FIES items indicates the severity of food insecurity associated with each raw score. The calibrations were estimated on a logit scale (with equal discrimination = 1), mean set to 0, and SD of 1,

^2:^ Infit, item-infit mean square statistic.

^3:^ Outfit, item-outfit mean square statistic.
